# The 2020 MELODI workshop on the effects of spatial and temporal variation in dose delivery

**DOI:** 10.1007/s00411-022-01002-3

**Published:** 2022-10-25

**Authors:** Balázs G. Madas, Andrzej Wojcik

**Affiliations:** 1 Environmental Physics Department, Centre for Energy Research, Budapest, Hungary; 2 Centre for Radiation Protection Research, MBW Department, Stockholm University, Svante Arrhenius väg 20C, 106 91 Stockholm, Sweden; 3 Institute for Biology, Jan Kochanowski University, Kielce, Poland

## Abstract

A key activity of MELODI is to organise annual European meetings where scientific results and future directions and strategies of relevant research are discussed. The annual meetings, previously organised solely under the auspices of MELODI are, since 2016, jointly organised by the European platforms and referred to as European Radiation Protection Weeks (ERPW). In addition to ERPW meetings, MELODI organises and finances annual workshops dedicated to specific topics. Outputs and recommendations from the meetings are published as review articles. The 2020 workshop focussed on one of the cross cutting topics: the effects of spatial and temporal variation in dose delivery on disease risk. The current issue of REBS includes five review articles from the workshop on the effects of spatial and temporal variation in dose delivery and this editorial is a short summary of their content.

MELODI (Multidisciplinary European Low Dose Initiative) is one of the six European Platforms dedicated to radiation protection research that, since 2020, jointly form the umbrella organisation MEENAS. The other five platforms are ALLIANCE (focusing on radioecology), NERIS (focusing on emergency management), EURADOS (focusing on dosimetry issues), EURAMED (focusing on medical exposures) and SHARE (focusing on social sciences/humanities). MELODI was founded already in 2010 with the aim to coordinate and promote research and long-term competence on effects and risks to human health associated with low-dose and low-dose rate exposures to ionizing radiation. MELODI considers low doses to be those where there remains substantial uncertainty on the magnitude of health risk. The history and progress of MELODI has been reviewed in several articles (Belli et al. 2011, 2015; Salomaa et al. 2017; Repussard 2018).

At the beginning of its activity, MELODI identified six key issues requiring investigation to better understand the health effects and risks associated with exposure to low doses:

- The shape of dose–response for cancer;
- Tissue sensitivities for cancer induction;
- Individual variability in cancer risk;
- The effects of radiation quality;
- Risks from internal radiation exposure;
- Risks of, and dose response relationships for, non-cancer diseases and hereditary effects.

MELODI’s strategic research agenda (SRA) working group is responsible for regular updates of the SRA. The current SRA is divided into two research topics and two cross cutting topics. The research topics relate to cancers and non-cancer diseases. The cross-cutting topics that are relevant to both of these disease categories are individual variation in risk and effects of spatial and temporal variation in dose delivery on disease risk. The research required to improve the evidence base for the research and cross-cutting topics includes mechanistic approaches and observational (epidemiological) studies that should integrate biological and molecular markers to improve health risk evaluation of radiation exposure. The current and former versions of the MELODI SRAs can be found on the MELODI website: www.melodi-online.eu.

A key activity of MELODI is to organise annual European meetings where scientific results and future directions and strategies of relevant research are discussed. The annual meetings, previously organised solely under the auspices of MELODI are, since 2016, jointly organised by the European platforms and referred to as European Radiation Protection Weeks (ERPW). In addition to ERPW meetings, MELODI organises and finances annual workshops dedicated to specific topics. Until today, five such workshops have been organised on topics listed below:

- Individual response to radiation, 2018 in Malta;
- Non cancer diseases, 2019 in Sitges, Spain;
- Effects of spatial and temporal variation in dose delivery (in collaboration with EURADOS), 2020 planned to be in Budapest, Hungary, but held virtual because of the COVID-19 pandemic;
- Adverse outcome pathways (in collaboration with EURADOS and ALLIANCE), 2021 in Paris, France, held virtual because of the COVID-19 pandemic;
- Transgenerational radiation effects (in collaboration with ALLIANCE and the ICRP), 2022 in Budapest, Hungary.

Outputs and recommendations from the meetings are published as review articles and these include (Seibold et al. 2020; Averbeck et al. 2020; Gomolka et al. 2020; Kalman and Oughton 2020; Kreuzer and Bouffler 2021; Ainsbury et al. 2021; Lumniczky et al. 2021; Pasqual et al. 2021; Tapio et al. 2021; Chauhan et al. 2021, 2022).

The 2020 workshop focussed on one of the cross cutting topics: the effects of spatial and temporal variation in dose delivery on disease risk. The simplest relationship between absorbed dose and the associated health risk can be found and estimated if the exposure is (i) acute, (ii) whole body, (iii) from an external source of radiation, (iv) with low linear energy transfer (LET), which results in spatially homogeneously distributed energy deposition events in the human body. Most of these properties are applicable to the exposure in case of risk estimates obtained from the Japanese atomic-bomb survivors, a study fundamental for radiation protection (Ozasa et al. 2019). Compared to this “reference” exposure, however, many relevant exposure scenarios including most internal exposures are significantly different in terms of the spatial and temporal variation in dose delivery resulting in protracted exposures and spatially inhomogeneous dose distributions.

Spatial inhomogeneity in dose delivery can be observed at different levels. At the level of the whole organism it leads to dose inhomogeneity in different organs. Inhomogeneity within an organ (or tissue) leads to a dose gradient within the organ. Inhomogeneous energy deposition can also be observed at the cellular level, a situation related to radiation quality. In the spirit of the terminology above, the expression of partial cell exposures could also be used. Considering that special relativity argues that space and time are inextricably connected (Einstein 1905), it is reasonable to focus also on the effects of dose rate, i.e., the effects of inhomogeneous dose distributions along time (partial time exposures, if we are to continue the game of words). Although this terminology may appear somewhat bizarre and far-fetched, it clearly shows the analogy between various inhomogeneities, and the motivation behind the structure of the workshop.

The current issue of REBS includes five review articles from the 2020 workshop on the effects of spatial and temporal variation in dose delivery. The workshop sessions were held on three successive afternoons and one morning from 17 to 20th November 2020. The sessions were (1) low dose rate effects, (2) internal exposures; (3) radiation quality and, (4) partial body exposures. All sessions, except the internal exposures, are summarised in one review article, and separate papers deal with radon exposure and radiopharmaceutical therapy with alpha-emitting radionuclides.

Pazzaglia et al. (2022) reviewed the effects of partial body exposures which are the norm for exposures in radiation therapy, diagnostic radiology, and in occupational settings. Assessment of doses and risks for radiotherapy-related cancer and cardiovascular diseases becomes increasingly relevant with new treatment modalities and improved life expectancy, while the biological mechanisms and clinical relevance of out-of-target effects are still poorly understood. Systemic radiation effects in non-targeted tissues have been experimentally demonstrated with the immune system shown as the main player. Radiation effects on extracellular vesicles including their secretion and bioactive cargo (especially miRNAs and proteins) have important roles, while it has to be seen whether these effects are detrimental by propagating radiation damage, or protective by promoting tissue repair. New models of dose calculation for healthy tissues and improvement of risk models including personalized risk assessment for cancer and non-cancer effects from radiotherapy applications are priorities for future research.

With particular attention paid on the highly heterogeneous dose distributions within the lungs, Madas et al. (2022) presented an overview on the state of the art in epidemiology, clinical observations, cell biology, dosimetry, and modelling related to radon exposure and its association with cancer. Priorities for future research were also identified including the question whether radon can cause other diseases than lung cancer, and to investigate radon-related health risks in children, where internal dosimetry and internal microdosimetry will play important roles. The better understanding of the combined effects of radon and smoking is another priority, which can be achieved by integrating clinical, pathological, and molecular oncology data to obtain a radon-associated signature. 3D cultures of primary human bronchial epithelial cells were identified as the simplest experimental systems, where the effects of heterogeneous dose distributions can be studied, and they can also help to identify new and corroborate existing biomarkers. These experimental systems can provide valuable input and validation data for biophysical models, which then can help to find quantitative links between experimental and epidemiological data.

An overview of generalized internal dosimetry in nuclear medicine was provided by Li et al. (2022) highlighting the need of consideration of the dose heterogeneity within organs at risk in case of cancer treatment with alpha-emitting radiopharmaceuticals. As an example, biodistribution and dose heterogeneities within bone tissues were presented upon treatment with ^223^Ra. Low dose research in general, research on the effects of spatial variation in dose delivery in particular can benefit from preclinical and clinical studies on radiopharmaceutical therapy. The results can provide valuable data for development and validation of biophysical models applicable in radiation protection research. The authors also presented recommendations in view of the priorities of MELODI and EURADOS including potential joint research efforts related to radiopharmaceutical therapy.

Baiocco et al. (2022) focussed on how the spatial heterogeneity in energy deposition determines the biological outcome either when radiation dose is delivered by different kinds of radiation or with different modalities (partial cell exposures). They presented an overview of both experimental (including microbeam experiments) and theoretical approaches (including track-structure simulations) showing that damage interaction in multiple scales affects the biological effects of radiation, i.e., several relevant spatial scales coexist for carcinogenesis related endpoints like viable mutations. They underlined that the power of micro- and nanodosimetry relies in the potential to characterize radiation quality using only measurable physical quantities, while the topology of the genomic material and its active role as a target also play important roles. It is expected that the effects of spatial and temporal variation in energy depositions cannot be fully disentangled, as the dynamical response to the damage also determines the biological outcome in the longer term.

With a focus on low LET radiation exposure and dose rates relevant for radiological protection, Lowe et al. (2022) presented a comprehensive assessment of radiation dose rate studies to date, including molecular, cellular, animal, and human studies with very informative tables on the exposed biological systems, the irradiation details, the recorded outcomes, definitive findings and references. Limits and advantages of each approach were discussed. The most compelling evidence for dose rate effects have been summarized, however, illustration of the dose rates in these studies shows that that few data are available outside of epidemiology for dose-rate levels pertinent for radiological protection. Regarding the future, the authors presented not just suggestions on research objectives, but also detailed recommendations on how to perform experimental and epidemiological studies. They concluded that integrating epidemiology and radiobiology approaches will be essential to make strong conclusions on the effect of dose rate.

A general conclusion of the articles is that a multidisciplinary approach is required to better understand the effects of spatial and temporal variation in dose delivery and to improve risk assessment for cancer and non-cancer diseases. While the reviews focussed on different topics of spatial and temporal inhomogeneities, the conclusions also showed that these aspects cannot be completely disentangled, not in the least because in many real exposure scenarios, inhomogeneities occur in all the three spatial scales defined here, while the dose rate is also different compared to the reference radiation exposure. Anyway, it is important to keep in mind that the spatial and temporal aspects of dose delivery are crucial in radiation research. As Hall and Giaccia (2012) clearly point out: 4 Gy dose from X-rays does not deliver more energy than one sip of warm tea or the energy needed to lift someone ~ 40 cm from the ground. Even in this case (called homogeneous exposure), the difference is made by the difference in the spatial variation and temporal variation in dose delivery.

Radiation protection quantifies the effects of dose inhomogeneities on health risk by introducing weighting factors (ICRP_103 2007). In fact, all weighting factors applied in radiation protection are related to the spatial and temporal variation in dose delivery. Radiation weighting factors are used to take account of the effects of inhomogeneous energy deposition events at the micrometre or cellular scale. Tissue weighting factors consider the problem of inhomogeneity occurring at the macroscopic scale in the order of centimetres or at the level of the organism. The role of dose rate as a modifier of radiation effects is reflected in the dose and dose rate effectiveness factor DDREF. What is not considered is the intra-organ inhomogeneity, which means that the same cancer risk is associated with any exposure resulting in a given amount of energy absorbed by a single target cell or distributed among all target cells of a given organ (Madas 2016).

Seeing the analogies between partial body exposures, partial organ exposures, radiation quality and dose rate effects may also help to find out how to move forward aspects of the radiation protection system. This includes the question whether the effects of spatial variations in dose delivery within an organ should be taken into account, and if yes—how. This question is also addressed by the RadoNorm project.

A note at the end: shortly before the workshop, Gabriele Babini from University of Pavia passed away unexpectedly. Gabriele was a young and talented radiation researcher, particularly interested in the problem of dose inhomogeneities. His expertise, enthusiasm, and his kind nature are greatly missed.
